# Associations of reproductive risk score and joint exposure to ambient air pollutants with chronic obstructive pulmonary disease: a cohort study in UK Biobank

**DOI:** 10.1265/ehpm.23-00155

**Published:** 2023-12-07

**Authors:** Xiaowen Liu, Ninghao Huang, Ming Jin, Zhenhuang Zhuang, Wenxiu Wang, Yimin Zhao, Xiaojing Liu, Nan Li, Tao Huang

**Affiliations:** 1 Department of Epidemiology & Biostatistics, School of Public Health, Peking University, Beijing 100191, China; 2 Institute of Reproductive and Child Health, Peking University/ Key Laboratory of Reproductive Health, National Health Commission of the People’s Republic of China, Beijing 100191, China; 3 Key Laboratory of Molecular Cardiovascular Sciences (Peking University), Ministry of Education, Beijing, China; 4 Center for Intelligent Public Health, Institute for Artificial Intelligence, Peking University, China

**Keywords:** Chronic obstructive pulmonary disease, Reproductive risk score, Air pollution score, Joint effect, UK Biobank

## Abstract

**Background:**

Reproductive risk factors and air pollution for developing chronic obstructive pulmonary disease (COPD) have been documented separately. However, the combined effects of overall reproductive risk status on COPD and the extent to which this can be impacted by air pollution are unknown. The aim of this study was to construct a reproductive risk score (RRS) and an air pollution score (APS) and assess independent and joint associations between the two with incident COPD risk.

**Methods:**

78,027 female participants aged 40–69 years without baseline COPD from UK Biobank recruited between 2006 to 2010 were included in this study. RRS was constructed by 17 women’s reproductive health-related items, and APS incorporating PM_2.5_, PM_2.5–10_, PM_10_, NO_2_, and NO_x_ was calculated to assess the joint exposure level. The outcome of the incident COPD was identified through the in-patient hospital register. The associations of RRS and APS with COPD were examined by Cox proportional hazards regression.

**Results:**

The risk of COPD reached its highest in the fourth quartile of the RRS (adjusted HR: 2.23, 95% CI: 1.76–2.82, *P* for trend < 0.001). A dose-response manner can also be observed between higher tertile APS with increased COPD risk and the highest risk was found in the third tertile of the APS (adjusted HR: 1.37, 95% CI: 1.19–1.58, *P* for trend < 0.001). The relative excess risk due to interaction (RERI) of 0.030 (95% CI: 0.012–0.048) showed additive interaction between RRS and APS on COPD was significant. In the joint analysis, the combinations of both higher RRS and APS signified higher incident COPD risk.

**Conclusion:**

High RRS and high APS were jointly associated with increased COPD risks in a dose-response pattern. Using comprehensive indicators to identify women’s reproductive risk factors, together with the control of air pollution, is effective for COPD prevention.

**Supplementary information:**

The online version contains supplementary material available at https://doi.org/10.1265/ehpm.23-00155.

## 1 Introduction

Chronic obstructive lung disease (COPD), a group of respiratory diseases characterized by reversible persistent airflow limitation, is one of the top causes of mortality and morbidity worldwide [1, 2]. It is a major public health problem that imposes a heavy economic burden worldwide [3]. COPD was once thought to be a disease that primarily affects men, with major risk factors including smoking, occupational exposure, air pollution, and biogenetic factors [2]. However, rates and mortality of COPD are now increasing more sharply in women than in men, leading us to consider gender differences in COPD pathogenesis [4].

Existing studies have reported associations between various female reproductive health indicators and COPD-related indicators, suggesting a stronger susceptibility to respiratory disease mediated by female hormonal mechanisms [5–7]. Since COPD outcomes are not necessarily associated with a single reproductive health factor, further integration of female reproductive health indicators is needed to study their combined effects. In addition, due to the gender differences in exposure scenarios to indoor air fine particle pollution from biomass fuels which affect women disproportionately, and due to the gender-specific occupational exposure to various clouds of dust [2, 7], we assessed the effects of air pollutants as a mixture in an integrated manner. However, few previous studies have designed comprehensive indicators to evaluate the association between fertility plus air pollution and COPD.

In this study, we constructed a reproductive risk score (RRS) composed of women’s reproductive health status and an air pollution score (APS) incorporating PM_2.5_, PM_2.5–10_, PM_10_, NO_2_, and NO_x_. This study aims to use the two comprehensive indexes to assess the separate and joint risk of incident COPD among the female population from the UK Biobank study.

## 2 Materials and methods

### 2.1 UK Biobank participants

The study included female participants in UK Biobank, a population-based prospective cohort study that recruited over 500,000 participants aged 40–69 years across 22 centers in England, Wales, and Scotland from 2006 to 2010 [8]. At baseline, social demographic information, early life exposure, medical history, and other health-related information were collected through touch-screen questionnaires. Information on health-related outcomes was collected through hospital admissions and death registers [8]. Available data can be acquired publicly in the UK Biobank’s Showcase (http://www.ukbiobank.ac.uk/). UK Biobank has approval from the North West Multicenter Research Ethical Committee (MREC) and all participants provided written informed consent. Researchers who wish to use the resource do not need separate ethics approval (unless re-contact with participants is required). Our analysis excluded participants with baseline COPD and with missing data related to RRS and residential air pollution, eventually including 78,027 eligible participants (Fig. S1).

### 2.2 Reproductive risk score

According to meticulous consideration and literature search, RRS was constructed by considering health-related pregnancy outcomes in the screening process, including sexual intercourse, menstruation, childbearing history, miscarriage, contraceptive use, and surgery of the genital tract. We excluded participants with pregnancy-induced hypertension and gestational diabetes due to their strong correlations with other reproductive factors (i.e. Spearman’s rank correlation coefficient > 0.7). We also excluded single variables with a small sample size (sample size < 100,000). As a result, we incorporated 17 questions from touch-screen questionnaires to construct RRS, including lifetime number of sexual partners [9], start/end of menstruation [10, 11], reproductive information (number of children [12], childbearing age [13, 14], the weight of first child [15]), surgery of the genital tract (hysterectomy and ovariectomy and removal date) [16, 17], abnormal pregnancy events (stillbirth, spontaneous miscarriages, terminations) [18] and contraceptive intake [19].

For each reproductive risk factor, participants with the risk response were assigned a score of 1 and otherwise were defined as 0 points. Questions with a progressive relationship were assigned to the respective scores. For example, in question FH4, “Have you ever had any stillbirths, spontaneous miscarriages or terminations?”, the number of “stillbirths, spontaneous miscarriages and terminations” in FH4A to FH4C, respectively, had progressive relationships with FH4, and so were assigned to a group with a score range of 0–3. Then, all component scores were summed to acquire a total score ranging from 0 to 16, with higher scores indicating higher reproductive risk. Furthermore, we defined RRS as four levels: low-risk group (RRS: 0–1); low-mediate risk group (RRS: 2–3); high-mediate risk group (RSS: 4–5); high-risk group (RSS: 6–13) [20].

### 2.3 Air pollution and APS

The Land Use Regression (LUR) model, as part of the European Study of Cohorts for Air Pollution Effects (ESCAPE), was created with the annual average concentrations of PM_2.5_, PM_10_, PM_2.5–10_, NO_2_, and NO_x_ for the year 2010 finished monitoring between January 2010 and January 2011 [21]. Air pollution estimates for the years 2005–2007 were derived from EU-wide air pollution maps which were modeled based on a LUR model for Europe [22]. Air pollution exposures in our analysis were linked to participants’ baseline records through residential addresses.

We used the annual concentration data of PM_2.5_, PM_2.5–10_, and NO_x_ for the year 2010, while data of both NO_2_ and PM_10_ were the averaged concentration for several years (2005, 2006, 2007, and 2010 for NO_2_, 2007, and 2010 for PM_10_). To access the joint exposure to the above air pollutants, we created a weighted APS by summing up concentrations of the five air pollutants, weighted by the multivariable-adjusted risk estimates (β coefficients) on COPD, with individual air pollutants as the independent variable [23]. The equation was: $\mathrm{APS}=(\beta_{{\text{PM}}_{2.5}}\times {\text{PM}}_{2.5}+\beta_{{\text{PM}}_{2.5\text{–}10}}\times {\text{PM}}_{2.5\text{–}10}+\beta_{{\text{PM}}_{10}}\times {\text{PM}}_{10}+\beta_{{\text{NO}}_{2}}\times {\text{NO}}_{2}+\beta_{{\text{NO}}_{x}}\times {\text{NO}}_{x})\times (5/\text{sum of the }\beta \text{ coefficients})$ . In this study, the values of $\beta_{{\text{PM}}_{2.5}}$ , $\beta_{{\text{PM}}_{2.5\text{–}10}}$ , $\beta_{{\text{PM}}_{10}}$ , $\beta_{{\text{NO}}_{2}}$ and $\beta_{{\text{NO}}_{x}}$ were 0.138, 0.007, 0.051, 0.018 and 0.010, respectively. The higher APS indicates greater exposure to ambient air pollution. We further divided participants into three groups based on tertiles of the APS.

### 2.4 Outcomes

Prevalent COPD at baseline was defined by self-report and the 9th revisions of the International Classification of Diseases (ICD-9) codes 490 to 492, 494, and 496, and incident COPD was defined by the 10th revisions of the International Classification of Diseases (ICD-10) codes J40 to J44, including information on admissions and diagnoses from 22 assessment centers across England, Wales, and Scotland [8, 24].

### 2.5 Covariates

Self-reported information on other covariates was obtained at baseline through the touchscreen questionnaire. Our analysis included sociodemographic characteristics (age at recruitment, education, and employment), lifestyle factors (smoking status, alcohol intake, diet, and physical activity level), and anthropometric measurements (body mass index). Levels of education included vocational, lower secondary, upper secondary, higher, and none of the above. Levels of employment included paid employment or self-employed work, not in paid employment, and retirement. Smoking status was classified as current, previous, and never. Healthy alcohol intake was defined as between 0 to 14 g/day for women. A healthy diet was defined as adherence to the following 4–5 ideal food groups: total fruit intake ≥ 3 servings/day; total vegetable intake ≥ 3 servings/day; total fish intake ≥ 2 servings/week; processed meat intake ≤ 1 serving/week; and unprocessed meat intake ≤ 1.5 servings/week. Healthy physical activity was defined as adherence to moderate activity ≥ 150 minutes/week or vigorous activity ≥ 75 minutes/week or equivalent combination. Body mass index (BMI) was calculated as weight divided by height squared (kg/m^2^) during the initial assessment centre visit.

### 2.6 Statistical analysis

We summarized the baseline characteristics according to descriptive statistics with the means (standard deviation, SD) for continuous variables and the number (percentage, %) for categorical variables. The basic characteristics of female participants in the different groups were compared using Student’s *t* test for quantitative variables and the χ^2^ test for categorical variables.

We used Cox proportional hazards regression to examine the associations of RRS, individual air pollutants, and the APS with COPD a total of 7 times, containing both categorical and continuous variables. Follow-up person-years were calculated for the duration from baseline at enrolment to the first occurrence of either the incidence date of COPD, loss of follow-up, death from other causes, or the end of follow-up (January 31, 2018) if the participants were still alive and disease-free. The multivariable models were adjusted for age, BMI, education, employment, smoking status, alcohol intake, healthy diet, and physical activity. Cox proportional hazards regression was also used to examine the joint association of RRS categories and APS quartiles with COPD risk, using participants who were in both the low risk RRS and APS group as the reference. The same set of covariates was used and the results were presented as a forest plot.

The additive interaction analysis between continuous variables of RRS and APS based on the Cox regression model was also examined, applying relative excess risk due to interaction (RERI) and attributable proportion (AP). The equations were: *RERI* = *HR*_++_ − *HR*_+−_ − *HR*_−+_ + 1 and *AP* = *RERI*/*HR*_++_.

Further in the stratified analysis, we analyzed multiplicative interactions using the likelihood ratio test between RRS and APS. Participants were classified by RRS and APS to estimate COPD risks with the Cox regression model adjusting for the same set of covariates, using participants who were in the low risk RRS group of each APS tertile as references.

In addition, we used the area under the receiver operator characteristic curve (AUC) and integrated discrimination improvement (IDI) to describe the performance of the RRS and ev aluate its discriminative ability [25, 26]. The AUC value ranges from 0 to 1, with higher numbers indicating better performance. IDI is one of the alternatives to AUC for assessing the ability of indexes to predict binary outcomes. Positive IDI with statistical significance indicates the improvement towards model prediction with the index.

All analyses were performed using STATA/SE version 16.0. Statistical significance was defined by P < 0.05 in two-sided test.

## 3 Results

Of the 78,027 female participants included in this study, a total of 1,361 (1.74%) incident COPD cases were documented at the end of the follow-up. The baseline characteristics of participants are summarized in Table 1. Participants with COPD were older, with higher BMI, and less likely to be highly educated or in paid employment compared with those without COPD. Besides, most of them were current or previous smokers, and fewer adhered to a healthy alcohol intake, diet, or physical activity. The mean estimates of PM_2.5_, PM_10_, NO_2_, NO_x_, and RRS were higher among participants with incident COPD (P < 0.001).

**Table 1:** Baseline characteristics of participants in the UK Biobank study according to COPD.

**Characteristics**	**Incident COPD**		***P* value**

	**Yes (N = 1,361)**	**No (N = 76,666)**	
Age (years, mean[SD])	62.19 (5.16)	60.37 (5.26)	<0.001
Body mass index (kg/m^2^, mean[SD])	28.10 (5.91)	27.08 (4.85)	<0.001
Education (%)			<0.001
Work-related practical qualifications	115 (8.59)	3,635 (4.80)	
Lower secondary education	342 (25.54)	21,980 (29.05)	
Upper secondary education	84 (6.27)	8,294 (10.96)	
Higher education	251 (18.75)	26,737 (35.34)	
None of the above	547 (40.85)	15,006 (19.84)	
Employment (%)			<0.001
In paid employment or self-employed	365 (26.98)	34,224 (44.93)	
Not in paid employment	148 (10.94)	5,320 (6.98)	
Retired	840 (62.08)	36,627 (48.09)	
Smoking states (%)			<0.001
Current	479 (35.38)	4,760 (6.23)	
Previous	582 (42.98)	25,354 (33.17)	
Never	293 (21.64)	46,324 (60.60)	
Healthy alcohol intake^a^ (%)	474 (34.85)	36,875 (48.11)	<0.001
Healthy diet^b^ (%)	165 (12.21)	13,474 (17.64)	<0.001
Healthy physical activity level (%)	786 (59.55)	53,346 (70.77)	<0.001
Reproductive risk score (mean[SD])	3.45 (1.75)	2.97 (1.52)	<0.001
PM_2.5_ (µg/m^3^, mean[SD])	10.17 (1.08)	9.88 (1.01)	<0.001
PM_2.5–10_ (µg/m^3^, mean[SD])	6.43 (0.88)	6.39 (0.89)	0.123
PM_10_ (µg/m^3^, mean[SD])	19.40 (1.81)	19.10 (1.83)	<0.001
NO_2_ (µg/m^3^, mean[SD])	30.17 (8.82)	28.09 (8.42)	<0.001
NO_X_ (µg/m^3^, mean[SD])	46.71 (18.98)	42.33 (14.57)	<0.001

^a^Healthy alcohol intake: 0 < women ≤ 14 g/day.

^b^Healthy diet: meeting 4–5 ideal food groups.

Abbreviation: COPD, chronic obstructive pulmonary disease; SD, standard deviation; PM_2.5_, particulate matter with aerodynamic diameter ≤2.5 µm; PM_2.5–10_, particulate matter with an aerodynamic diameter between 2.5 and 10 µm; PM_10_, particulate matter with aerodynamic diameter ≤10 µm; NO_2_, nitrogen dioxide; NOx, nitrogen oxide.

Associations between RRS, individual air pollutants, and APSs with COPD were shown in Table 2 and Table S1. The trend analysis revealed that higher RRS was associated with an increased risk of COPD in the multivariable-adjusted models (HR: 1.15, 95% CI: 1.12–1.19, P trend < 0.001). Compared to the low RRS group, the adjusted HRs (95% CI) of COPD for participants in the low-mediate, high-mediate, and high RRS groups were 1.25 (1.04, 1.51), 1.59 (1.30, 1.93) and 2.23 (1.76, 2.82), respectively. PM_2.5_, PM_10_, NO_2_, and NO_x_ were each associated with an increased risk of COPD in a dose-response manner after multivariate adjustment (P trend < 0.001). With the lowest tertile (Q1) as the reference, the higher tertile of PM_2.5_, PM_10_, NO_2_, and NO_x_ had higher risks of COPD. The adjusted HRs (95%CI) in Q2 were 1.05 (0.91, 1.22), 1.11 (0.96, 1.28), 1.08 (0.93, 1.26), and 1.16 (1.00, 1.34); and the effects were 1.31 (1.14, 1.50), 1.29 (1.12, 1.48), 1.45 (1.26, 1.67) and 1.29 (1.12, 1.49) in Q3, respectively. Considering the joint exposure of the 5 air pollutants, a dose-response manner can be observed in the association between a higher tertile APS and increased risk of COPD (P trend < 0.001). The adjusted HRs (95%CI) when the higher tertile was compared with the lowest tertile (Q1) were 1.08 (0.93, 1.25) and 1.37 (1.19, 1.58), respectively. Table S1 demonstrated that the associations of covariates with COPD were consistent across multivariable-adjusted models in RRS, APS, and air pollutants. The risk of COPD increased with older age and more BMI. To be not in paid employment, in retirement and smoking were risk factors for COPD and healthy alcohol intake, healthy diet, healthy physical activity level, and higher educational qualifications were protective factors.

**Table 2:** HRs (95% CI) for RRS and air pollution with the risk of incident COPD.

	**COPD/No. (%)**	**HR (95%CI)**	

		**Crude**	**Multivariable adjusted^a^**
**Reproductive risk score**			
The low risk RRS (0–1)	150/12,216 (1.23)	Ref.	Ref.
The low-mediate RRS (2–3)	617/40,420 (1.53)	1.25 (1.04, 1.49)	1.25 (1.04, 1.51)
The high-mediate risk RRS (4–5)	431/20,549 (2.10)	1.72 (1.43, 2.08)	1.59 (1.30, 1.93)
The high risk RRS (6–13)	163/4,842 (3.37)	2.79 (2.24, 3.49)	2.23 (1.76, 2.82)
Each point increment		1.21 (1.17, 1.25)	1.15 (1.12, 1.19)
*P* for trend		<0.001	<0.001
**PM_2.5_ tertiles** (µg/m^3^)			
Q1 (8.17–9.45)	348/26,009 (1.34)	Ref.	Ref.
Q2 (9.45–10.22)	413/26,009 (1.59)	1.19 (1.03, 1.38)	1.05 (0.91, 1.22)
Q3 (10.22–21.31)	600/26,009 (2.31)	1.72 (1.51, 1.96)	1.31 (1.14, 1.50)
Each SD increment		1.28 (1.23, 1.35)	1.15 (1.09, 1.21)
*P* for trend*		<0.001	<0.001
**PM_2.5–10_ tertiles** (µg/m^3^)			
Q1 (5.57–5.90)	406/26,009 (1.56)	Ref.	Ref.
Q2 (5.90–6.36)	468/26,009 (1.80)	1.17 (1.02, 1.33)	1.11 (0.96, 1.27)
Q3 (6.36–11.24)	487/26,009 (1.87)	1.21 (1.06, 1.38)	1.09 (0.95, 1.25)
Each SD increment		1.04 (0.99, 1.09)	1.01 (0.95, 1.06)
*P* for trend*		0.013	0.378
**PM_10_ tertiles** (µg/m^3^)			
Q1 (12.87–18.35)	362/26,009 (1.39)	Ref.	Ref.
Q2 (18.35–19.73)	459/26,009 (1.76)	1.27 (1.11, 1.46)	1.11 (0.96, 1.28)
Q3 (19.73–29.41)	540/26,009 (2.08)	1.50 (1.32, 1.72)	1.29 (1.12, 1.48)
Each SD increment		1.18 (1.12, 1.24)	1.10 (1.04, 1.16)
*P* for trend*		<0.001	<0.001
**NO_2_ tertiles** (µg/m^3^)			
Q1 (9.04–24.12)	331/26,009 (1.27)	Ref.	Ref.
Q2 (24.12–30.58)	433/26,009 (1.66)	1.31 (1.14, 1.51)	1.08 (0.93, 1.26)
Q3 (30.58–107.81)	597/26,009 (2.30)	1.84 (1.61, 2.11)	1.45 (1.26, 1.67)
Each SD increment		1.25 (1.20, 1.32)	1.17 (1.11, 1.23)
*P* for trend*		<0.001	<0.001
**NO_X_ tertiles** (µg/m^3^)			
Q1 (19.74–36.03)	336/26,009 (1.29)	Ref.	Ref.
Q2 (36.03–45.89)	450/26,009 (1.73)	1.35 (1.17, 1.56)	1.16 (1.00, 1.34)
Q3 (45.89–252.10)	575/26,009 (2.21)	1.72 (1.51, 1.97)	1.29 (1.12, 1.49)
Each SD increment		1.24 (1.19, 1.28)	1.16 (1.11, 1.21)
*P* for trend*		<0.001	<0.001
**Air Pollution Score tertiles**			
Q1 (48.94–69.57)	338/26,009 (1.30)	Ref.	Ref.
Q2 (69.57–77.50)	423/26,009 (1.63)	1.26 (1.09, 1.45)	1.08 (0.93, 1.25)
Q3 (77.50–177.66)	600/26,009 (2.31)	1.79 (1.57, 2.05)	1.37 (1.19, 1.58)
Each SD increment		1.29 (1.23, 1.35)	1.17 (1.11, 1.24)
*P* for trend*		<0.001	<0.001

^a^Adjusted for age, body mass index, education, employment, alcohol drinking, smoking status, healthy diet status and physical activity.

*Linear trend across categories was quantified by assigning median value to each category and modelling this variable as continuous variable.

Abbreviation: HR, hazard ratio; CI, confidence interval; COPD, chronic obstructive pulmonary disease; RRS, reproductive risk score; SD, standard deviation; PM_2.5_, particulate matter with aerodynamic diameter ≤2.5 µm; PM_2.5–10_, particulate matter with an aerodynamic diameter between 2.5 and 10 µm; PM_10_, particulate matter with aerodynamic diameter ≤10 µm; NO_2_, nitrogen dioxide; NOx, nitrogen oxide.

**Fig. 1:**
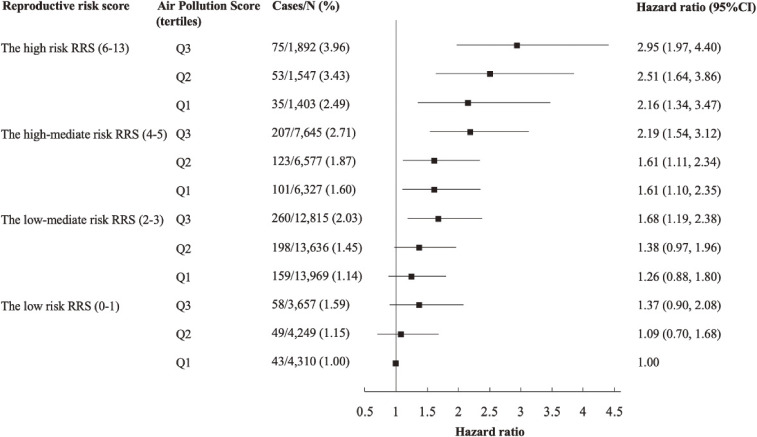
The joint association of RRS categories and APS tertiles with the risk of COPD. Hazard Ratios (95% CIs) were estimated with Cox proportional hazard models adjusted for age, body mass index, education, employment, smoking status, alcohol drinking, healthy diet status, and physical activity. RRS, reproductive risk score; APS, air pollution score; COPD, chronic obstructive lung disease; CI, confidence interval.

We further explored the stratified and joint analysis, as well as the interaction of RRS and APS with the occurrence of COPD. With the low risk RRS group as a reference, the adjusted HR (95% CI) for the low-mediate, high-mediate, and high risk RRS group in the Q3 tertile APS group were 1.21 (0.90, 1.63), 1.55 (1.14, 2.11), and 2.00 (1.39, 2.89) in Table S2, respectively. Similar results were found in the other two APS groups. Individuals had an increased risk of COPD with the increased APS levels in a dose-response pattern in the respective RRS risk group. The adjusted HR (95% CI) of RRS-COPD also showed a slight increase with the elevated APS. From Q1 APS to Q3 APS, the adjusted HRs (95% CI) of COPD with per score increment of RRS were 1.14 (1.06, 1.22), 1.15 (1.08, 1.22), and 1.15 (1.10, 1.21), respectively. Figure 1 shows the joint association of different categories of RRS and APS tertiles on the incident risk of COPD. We classified the participants according to the joint categories of RRS and APS, with the group of low RRS (0–1) and lowest APS tertile as the reference. The trend of incident COPD risk in the respective RRS subgroups gradually increased with the elevated APS tertiles. Participants with the highest RRS (6–13) and in the highest APS tertile group (Q3) had the highest risk of COPD (HR 2.95, 95% CI 1.97 to 4.40).

We also detected additive interaction effects between RRS and APS on the risks of incident COPD in Table 3. The RERI for COPD was 0.030 (95% CI: 0.012, 0.048; P = 0.001). However, no significant multiplicative interaction was found between RRS and APS in COPD (*P* for interaction is 0.086; Table S2).

**Table 3:** Attributing effects to additive interaction between RRS and APS on the risk of COPD.

**Additive Interaction**	**Crude**		**Multivariable adjusted^a^**	

	**Estimate^b^ (95% CI)**	***P* value**	**Estimate^b^ (95% CI)**	***P* value**
Relative excess risk due to interaction	0.059 (0.039, 0.079)	<0.001	0.030 (0.012, 0.048)	0.001
Attributable proportion for reproductive risk score	0.348 (0.259, 0.437)	<0.001	0.486 (0.222, 0.751)	<0.001
Attributable proportion for air pollution score	0.532 (0.408, 0.655)	<0.001	0.380 (−0.075, 0.835)	0.996
Attributable proportion for addictive interaction	0.120 (0.056, 0.185)	<0.001	0.134 (−0.104, 0.372)	0.271

^a^Adjusted for age, body mass index, education, employment, alcohol drinking, smoking status, healthy diet status and physical activity.

^b^Relative excess risk due to interaction and attributable proportions were estimated based on hazard ratio and proportion hazard ratio, respectively.

Abbreviation: RRS, reproductive risk score; APS, air pollution score; COPD, chronic obstructive pulmonary disease; CI, confidence interval

The performance of the RRS and integrated discrimination improvements on COPD can be observed in Table S3. Both AUC of each item of RRS and comprehensive RRS were higher than 0.8, which indicated that each item of RRS and comprehensive RRS had a good performance in predicting COPD. The result of IDI% showed the improved discriminating ability of comprehensive RRS relative to any single item of RRS.

## 4 Discussion

In this study, we aimed to construct two comprehensive scores to assess the association between reproductive risk factors plus ambient air pollution and COPD in the UK Biobank cohort. The results showed that the grades of RRS showed a significantly positive dose-response relationship with the risk of developing COPD. We also found a positive association between air pollution fractions consisting of long-term exposure to air pollutants including PM_2.5_, PM_2.5–10_, PM_10_, NO_2_, and NO_x_ and the incidence of COPD in women. In addition, the association between RRS and COPD incidence was significantly modified by APS. COPD risk with joint effect of RRS and APS exceeded the sum of the individual effects of the two factors (RERI: 0.030, 95% CI: 0.012, 0.048). More attention should be paid to public health measures to reduce COPD risk among women exposed to both reproductive risk and ambient air pollution factors.

As the prevalence and mortality of COPD in women have increased in recent years, more and more studies have begun to explore COPD risk factors specific to women. The association between multiple female reproductive health factors across the life course and COPD has been confirmed. A previous study of women in the UK Biobank [5] showed that parity >3, late menarche (>15 years) or early menopause (<47 years), hysterectomy, and bilateral oophorectomy were associated with a higher risk of COPD-related hospitalization/death. In contrast, the use of oral contraceptives reduced the time of COPD-related hospitalization/mortality and was associated with improved FEV1/FVC lung function indicators, possibly through altered hormone levels that improved lung function in postmenopausal women [5]. Several large epidemiological studies have shown that the menstrual cycle causes fluctuations in lung function among women with airway disease, suggesting that estradiol and progesterone in the menstrual cycle may be involved in the occurrence of COPD [27]. Evidence from population-based studies suggested that ambient air pollution contributes more to the burden of COPD than previously recognized [28]. Our results similarly confirmed evidence from previous studies on the association between exposure to different air pollutants and COPD risk. Surveillance data from southwest China demonstrated that PM_2.5_ and NO_2_ increased COPD-related mortality by 2.7% (95% CI 1.0–4.4%) and 3.6% (95% CI 1.7–5.6%) respectively, in the elderly population aged 60 years and older [29]. A continuous cross-sectional study of 4,757 women in Germany found that COPD and lung function were most affected by particulate matter and traffic-related exposures to PM_10_ [30, 31]. Another study involving 57,053 participants in Denmark reported that the development of COPD was associated with 35-year average NO_2_ and NO_x_ levels, during which NO_2_ had the strongest effect [32]. What’s more, the covariates significantly associated with COPD in our multivariable-adjusted Cox model implied effective control for confounders. Previous studies suggested that on average 80–90% of COPD patients were primarily caused by smoking and that the harmful effects of cigarette smoke persisted even after quitting [33, 34]. We also found that HRs for current smokers are almost four times higher than for previous smokers, using never smokers as a reference. Aligning with our results, previous studies suggested that COPD patients had lower employment rates and lower educational level compared to controls, however the causality needs further discussion [35–37]. Previous studies have also consistently revealed significant effects of alcohol consumption, diet, and physical activity on COPD, emphasizing the importance of healthy lifestyle choices in reducing COPD risk [38–40].

The following possible mechanisms may explain the positive correlations between female reproductive factors plus air pollution and COPD. There are differences in the histological patterns of COPD between men and women, of which women are more likely to have small-airway COPD than emphysema [41, 42]. The ovariectomized female mice developed the same pattern of COPD as male mice, suggesting that estrogen is responsible for the sex difference [43]. In addition, physiological structure differences also contribute to the susceptibility of women to COPD. Studies have found that women’s airways are relatively smaller than men’s for the same vital capacity, so the concentration of pollutants per unit area of the surface of small airways may be higher [42]. COPD associated with air pollution also showed more small airways, which may reflect the predominance of women in the risk factor of air pollutants [44]. The pathophysiological effects of air pollution on COPD and the underlying mechanisms are not fully understood, and so far hypotheses including oxidative damage, inflammation damage, and DNA damage have been proposed [45]. Acute exposure to PM_2.5_ can lead to infiltration and hyperemia of inflammatory cells in lung tissue, which are phagocytosed by lung macrophages after inhalation and stimulate the release of inflammatory factors [45–48]. PM can produce oxygen free radicals, which can stimulate cells to produce a large number of reactive oxygen species (ROS) after inhalation. Oxidative stress induced by ROS may play a key role in driving COPD-related inflammation with a dose-dependent relationship in a certain concentration range [45, 46]. The combined effect of the interaction of multiple air pollutants makes the relevant mechanisms more complex and may cause acute exacerbation of COPD [49].

Significant additive interaction and joint correlation between RRS and APS on the risk of COPD indicated that combined exposure to air pollution can increase the risk of COPD due to reproductive risk factors, and the interaction of the two factors is greater than the sum of their effects alone. Although no significant multiplicative interaction was observed, there is a theory indicating that the biological interaction between the two factors should be assessed as additive rather than multiplicative if both are responsible for the disease [50]. This biological interaction can be explained to some extent by the effect of the overlap of reproductive factors and air pollution on COPD-related mechanisms. As mentioned above, the accelerated progression of COPD in women is mainly determined by hormones-related small airway fibrosis [42]. Ambient air pollution may exacerbate the hormonal imbalance caused by reproductive risk factors in women and increase COPD susceptibility. For women with long-term exposure to air pollutants, female hormones, most notably estrogen, may accelerate the metabolism of pollutants and produce more strong oxidants through estrogen regulation of cytochrome P450 (CYP) expression, such as N-nitroso derivatives and metabolites related to polycyclic aromatic hydrocarbons (PAHs), leading to increased oxidative stress in the lungs of these women [51]. Therefore, hormone replacement therapy is often used to improve lung function to reduce the risk of airflow obstruction and bronchial hyperresponsiveness in postmenopausal women [52, 53]. Above all, early intervention of reproductive risk factors and decrease of air pollution exposure can reduce the risk of COPD development. We constructed the RRS composite score to identify reproduction-related risks in individuals, the APS score to synthetically assess exposure to multiple pollutants, and predict the risk of COPD in individuals, which can serve to prevent and reduce the COPD burden at the population level.

In this study, we have constructed comprehensive scores to prospectively examine the association of reproductive health factors and environmental pollution factors with COPD in UK Biobank data. However, there are still some limitations to our study. First, only 28.5% of female participants in UK Biobank had data on reproductive risk factors, and there might be confounding factors not included in the adjustment in subsequent analyses, resulting in some degree of baseline variation. We found that the basic characteristics of women in the final analysis (n = 78,027) differed from those of women excluded from original cohort (n = 273,396) except BMI. It may be not appropriate to generalize our results to other populations with different basic characteristics. Second, during the construction of the RRS, we did not include reproductive risk-related conditions including hypertensive disorders of pregnancy and gestational diabetes. Finally, air pollutants such as O_3_, sulfur dioxide, and carbon monoxide were not available in UK Biobank, and we also did not have relevant data about air pollution dynamic changes during follow-up. Therefore, further studies are needed to confirm our inferences and explore relevant mechanisms of action.

## 5 Conclusion

The results of this large prospective cohort study suggested that the higher risk RRS and the higher APS were both significantly associated with an increased risk of incident COPD among female participants. There is also an interaction between the two risk factors, which showed higher levels of air pollution exposure may increase the risk of incident COPD due to reproductive risk factors. Our findings demonstrated the importance of intervention as early as possible in high reproductive risk groups, and comprehensively controlling air pollution for the prevention of COPD in women, which may generally reduce the socioeconomic burden caused by COPD.

## Abbreviations

COPD: Chronic obstructive lung disease
RRS: reproductive risk score
APS: air pollution score
RERI: the relative excess risk due to interaction

## Supplementary information

**Additional file 1:** **Figure S1** Flow diagram.

**Additional file 2:** **Table S1.** HRs (95% CI) for covariates in the multivariable adjusted Cox regression model. **Table S2.** Stratified analysis on incident COPD risk by APS tertiles in UK Biobank. **Table S3.** AUC and IDI of COPD according to individual items of and total RRS.

## Declarations

### Availability of data and materials

All data analyzed in this paper are available from the corresponding author upon reasonable request.

## References

[1] Ho T, . Under- and over-diagnosis of COPD: a global perspective. Breathe (Sheff). 2019;15(1):24–35. 30838057 10.1183/20734735.0346-2018 PMC6395975

[2] Viegi G, . Definition, epidemiology and natural history of COPD. Eur Respir J. 2007;30(5):993–1013. 17978157 10.1183/09031936.00082507

[3] Lopez-Campos JL, Tan W, Soriano JB. Global burden of COPD. Respirology. 2016;21(1):14–23. 26494423 10.1111/resp.12660

[4] Aryal S, Diaz-Guzman E, Mannino DM. COPD and gender differences: an update. Transl Res. 2013;162(4):208–18. 23684710 10.1016/j.trsl.2013.04.003

[5] Tang R, Fraser A, Magnus MC. Female reproductive history in relation to chronic obstructive pulmonary disease and lung function in UK biobank: a prospective population-based cohort study. BMJ Open. 2019;9(10):e030318. 10.1136/bmjopen-2019-030318 PMC6830692 31662371

[6] Townsend EA, Miller VM, Prakash YS. Sex differences and sex steroids in lung health and disease. Endocr Rev. 2012;33(1):1–47. 22240244 10.1210/er.2010-0031 PMC3365843

[7] Varkey AB. Chronic obstructive pulmonary disease in women: exploring gender differences. Curr Opin Pulm Med. 2004;10(2):98–103. 15021178 10.1097/00063198-200403000-00003

[8] Sudlow C, . UK biobank: an open access resource for identifying the causes of a wide range of complex diseases of middle and old age. PLoS Med. 2015;12(3):e1001779. 25826379 10.1371/journal.pmed.1001779 PMC4380465

[9] Mercer CH, . Changes in sexual attitudes and lifestyles in Britain through the life course and over time: findings from the National Surveys of Sexual Attitudes and Lifestyles (Natsal). Lancet. 2013;382(9907):1781–94. 24286784 10.1016/S0140-6736(13)62035-8 PMC3899021

[10] Brooks-Gunn J, Warren MP, Hamilton LH. The relation of eating problems and amenorrhea in ballet dancers. Med Sci Sports Exerc. 1987;19(1):41–4. 3469489

[11] Shifren JL, Gass ML, N.R.f.C.C.o.M.W.W. Group. The North American Menopause Society recommendations for clinical care of midlife women. Menopause. 2014;21(10):1038–62. 25225714 10.1097/GME.0000000000000319

[12] Foldspang A, . Parity as a correlate of adult female urinary incontinence prevalence. J Epidemiol Community Health. 1992;46(6):595–600. 1494074 10.1136/jech.46.6.595 PMC1059675

[13] Socolov DG, . Pregnancy during Adolescence and Associated Risks: An 8-Year Hospital-Based Cohort Study (2007–2014) in Romania, the Country with the Highest Rate of Teenage Pregnancy in Europe. Biomed Res Int. 2017;2017:9205016. 28133615 10.1155/2017/9205016 PMC5241487

[14] Bayrampour H, Heaman M. Advanced maternal age and the risk of cesarean birth: a systematic review. Birth. 2010;37(3):219–26. 20887538 10.1111/j.1523-536X.2010.00409.x

[15] Juul F, . Birth weight, early life weight gain and age at menarche: a systematic review of longitudinal studies. Obes Rev. 2017;18(11):1272–88. 28872224 10.1111/obr.12587

[16] Nitecki R, . Survival After Minimally Invasive vs Open Radical Hysterectomy for Early-Stage Cervical Cancer A Systematic Review and Meta-analysis. JAMA Oncol. 2020;6(7):1019–27. 32525511 10.1001/jamaoncol.2020.1694 PMC7290695

[17] Orozco LJ, . Hysterectomy versus hysterectomy plus oophorectomy for premenopausal women. Cochrane Database Syst Rev. 2014;(7). 10.1002/14651858.CD005638.pub3 PMC7388914 25101365

[18] du Foss NA, . Advanced paternal age is associated with an increased risk of spontaneous miscarriage: a systematic review and meta-analysis. Hum Reprod Update. 2020;26(5):650–69. 32358607 10.1093/humupd/dmaa010 PMC7456349

[19] Gierisch JM, . Oral Contraceptive Use and Risk of Breast, Cervical, Colorectal, and Endometrial Cancers: A Systematic Review. Cancer Epidemiol Biomarkers Prev. 2013;22(11):1931–43. 24014598 10.1158/1055-9965.EPI-13-0298

[20] Zhao Y, . Associations of polysocial risk score, lifestyle and genetic factors with incident type 2 diabetes: a prospective cohort study. Diabetologia. 2022;65(12):2056–65. 35859134 10.1007/s00125-022-05761-y

[21] Eeftens M, . Development of Land Use Regression models for PM(2.5), PM(2.5) absorbance, PM(10) and PM(coarse) in 20 European study areas; results of the ESCAPE project. Environ Sci Technol. 2012;46(20):11195–205. 22963366 10.1021/es301948k

[22] Vienneau D, . Western European land use regression incorporating satellite- and ground-based measurements of NO_2_ and PM_10_. Environ Sci Technol. 2013;47(23):13555–64. 24156783 10.1021/es403089q

[23] Wang M, . Joint exposure to various ambient air pollutants and incident heart failure: a prospective analysis in UK Biobank. Eur Heart J. 2021;42(16):1582–91. 33527989 10.1093/eurheartj/ehaa1031 PMC8060055

[24] Palmer LJ. UK Biobank: bank on it. Lancet. 2007;369(9578):1980–2. 17574079 10.1016/S0140-6736(07)60924-6

[25] Wu J, . A nomogram for predicting overall survival in patients with low-grade endometrial stromal sarcoma: a population-based analysis. Cancer Commun. 2020;40(7):301–12. 10.1002/cac2.12067 PMC7365459 32558385

[26] Kerr KF, . Evaluating the incremental value of new biomarkers with integrated discrimination improvement. Am J Epidemiol. 2011;174(3):364–74. 21673124 10.1093/aje/kwr086 PMC3202159

[27] Han MK. Chronic Obstructive Pulmonary Disease in Women: A Biologically Focused Review with a Systematic Search Strategy. Int J Chron Obstruct Pulmon Dis. 2020;15:711–21. 32280209 10.2147/COPD.S237228 PMC7132005

[28] Salvi SS, Barnes PJ. Chronic obstructive pulmonary disease in non-smokers. Lancet. 2009;374(9691):733–43. 19716966 10.1016/S0140-6736(09)61303-9

[29] Chen J, . Effects of short-term exposure to ambient airborne pollutants on COPD-related mortality among the elderly residents of Chengdu city in Southwest China. Environ Health Prev Med. 2021;26(1):1–10. 33435864 10.1186/s12199-020-00925-x PMC7805042

[30] Schikowski T, . Long-term air pollution exposure and living close to busy roads are associated with COPD in women. Respir Res. 2005;6:152. 16372913 10.1186/1465-9921-6-152 PMC1352358

[31] Schikowski T, . Decline in air pollution and change in prevalence in respiratory symptoms and chronic obstructive pulmonary disease in elderly women. Respir Res. 2010;11:113. 20727210 10.1186/1465-9921-11-113 PMC2936381

[32] Andersen ZJ, . Chronic obstructive pulmonary disease and long-term exposure to traffic-related air pollution: a cohort study. Am J Respir Crit Care Med. 2011;183(4):455–61. 20870755 10.1164/rccm.201006-0937OC

[33] Yoshida T, Tuder RM. Pathobiology of cigarette smoke-induced chronic obstructive pulmonary disease. Physiol Rev. 2007;87(3):1047–82. 17615396 10.1152/physrev.00048.2006

[34] Hikichi M, . Pathogenesis of chronic obstructive pulmonary disease (COPD) induced by cigarette smoke. J Thorac Dis. 2019;11(Suppl 17):S2129. 31737341 10.21037/jtd.2019.10.43 PMC6831915

[35] Rai K, . Systematic review: chronic obstructive pulmonary disease and work-related outcomes. Occup Med. 2018;68(2):99–108. 10.1093/occmed/kqy012 29528460

[36] Kanervisto M, . Low socioeconomic status is associated with chronic obstructive airway diseases. Respir Med. 2011;105(8):1140–6. 21459567 10.1016/j.rmed.2011.03.008

[37] Murakami K, Kuriyama S, Hashimoto H. Economic, cognitive, and social paths of education to health-related behaviors: evidence from a population-based study in Japan. Environ Health Prev Med. 2023;28:9. 36709974 10.1265/ehpm.22-00178 PMC9884565

[38] Scoditti E, . Role of diet in chronic obstructive pulmonary disease prevention and treatment. Nutrients. 2019;11(6):1357. 31208151 10.3390/nu11061357 PMC6627281

[39] Pedersen BK, Saltin B. Evidence for prescribing exercise as therapy in chronic disease. Scand J Med Sci Sports. 2006;16(S1):3–63. 16451303 10.1111/j.1600-0838.2006.00520.x

[40] Kaluza J, . Alcohol consumption and risk of chronic obstructive pulmonary disease: a prospective cohort study of men. Am J Epidemiol. 2019;188(5):907–16. 30877760 10.1093/aje/kwz020

[41] Dransfield MT, . Gender differences in the severity of CT emphysema in COPD. Chest. 2007;132(2):464–70. 17573503 10.1378/chest.07-0863

[42] Barnes PJ. Sex Differences in Chronic Obstructive Pulmonary Disease Mechanisms. Am J Respir Crit Care Med. 2016;193(8):813–4. 27082528 10.1164/rccm.201512-2379ED

[43] Tam A, . Sex Differences in Airway Remodeling in a Mouse Model of Chronic Obstructive Pulmonary Disease. Am J Respir Crit Care Med. 2016;193(8):825–34. 26599602 10.1164/rccm.201503-0487OC

[44] Assad NA, . Chronic obstructive pulmonary disease secondary to household air pollution. Semin Respir Crit Care Med. 2015;36(3):408–21. 26024348 10.1055/s-0035-1554846

[45] Duan RR, Hao K, Yang T. Air pollution and chronic obstructive pulmonary disease. Chronic Dis Transl Med. 2020;6(4):260–9. 33336171 10.1016/j.cdtm.2020.05.004 PMC7729117

[46] Barnes PJ. Inflammatory mechanisms in patients with chronic obstructive pulmonary disease. J Allergy Clin Immunol. 2016;138(1):16–27. 27373322 10.1016/j.jaci.2016.05.011

[47] Chen B, Kan H. Air pollution and population health: a global challenge. Environ Health Prev Med. 2008;13:94–101. 19568887 10.1007/s12199-007-0018-5 PMC2698272

[48] Li R, . Effects of ambient PM_2.5_ on pathological injury, inflammation, oxidative stress, metabolic enzyme activity, and expression of c-fos and c-jun in lungs of rats. Environ Sci Pollut Res Int. 2015;22(24):20167–76. 26304807 10.1007/s11356-015-5222-z

[49] Sint T, Donohue JF, Ghio AJ. Ambient air pollution particles and the acute exacerbation of chronic obstructive pulmonary disease. Inhal Toxicol. 2008;20(1):25–9. 18236218 10.1080/08958370701758759

[50] Knol MJ, . Estimating interaction on an additive scale between continuous determinants in a logistic regression model. Int J Epidemiol. 2007;36(5):1111–8. 17726040 10.1093/ije/dym157

[51] Sin DD, . Understanding the biological differences in susceptibility to chronic obstructive pulmonary disease between men and women. Proc Am Thorac Soc. 2007;4(8):671–4. 18073400 10.1513/pats.200706-082SD

[52] Carlson CL, . Hormone replacement therapy is associated with higher FEV1 in elderly women. Am J Respir Crit Care Med. 2001;163(2):423–8. 11179117 10.1164/ajrccm.163.2.2003040

[53] Mueller JE, . Association of hormone replacement therapy with bronchial hyper-responsiveness. Respir Med. 2003;97(8):990–2. 12924529 10.1016/s0954-6111(03)00128-8

