# Supplementary figures and images for: Associations of reproductive risk score and joint exposure to ambient air pollutants with chronic obstructive pulmonary disease: a cohort study in UK Biobank
Source: Environ Health Prev Med. 2023 Dec 7;28:76. doi: 10.1265/ehpm.23-00155 (PMC10711373; doi:10.1265/ehpm.23-00155)

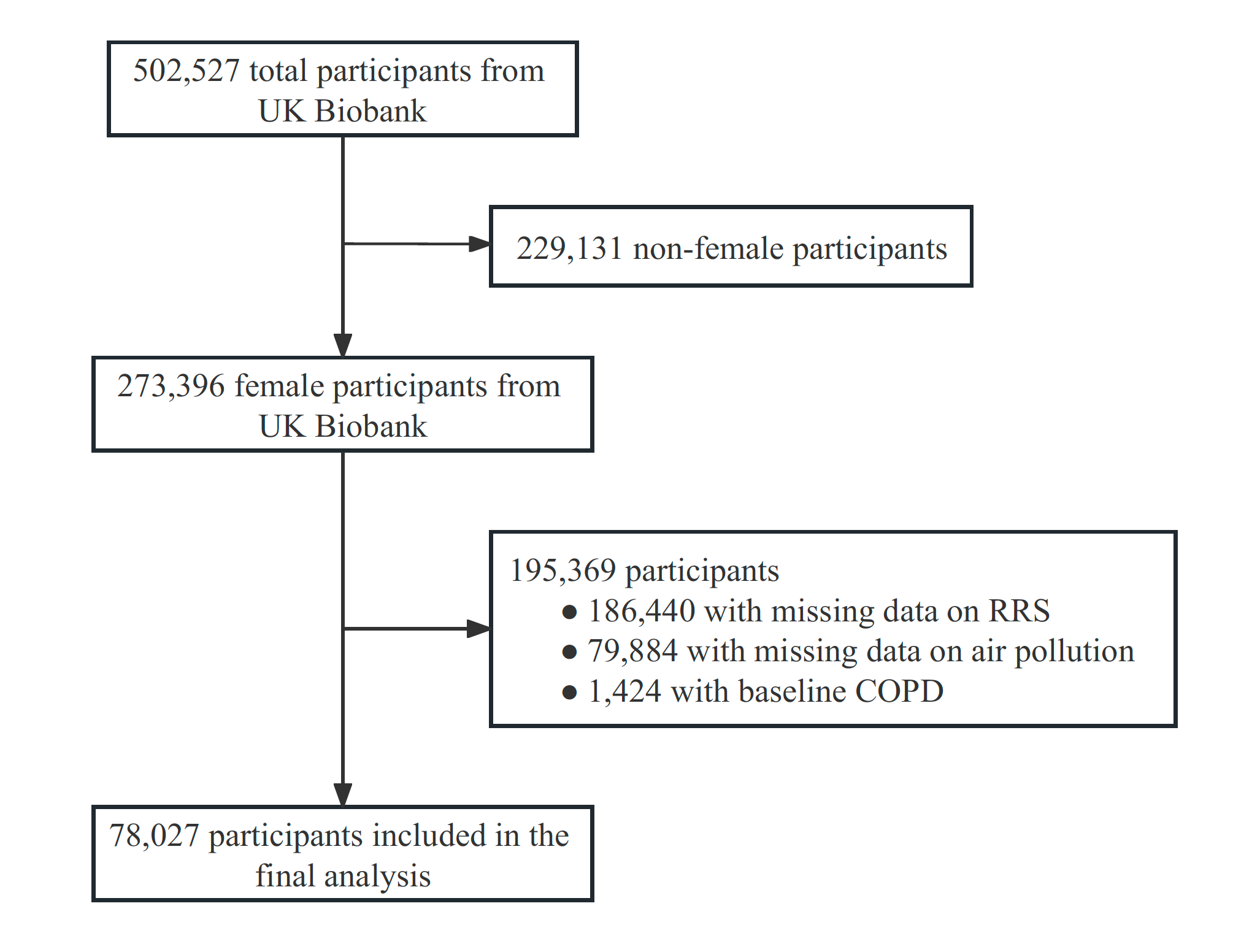

Supplement: Supplementary file 1 — Additional file 1: Figure S1 Flow diagram. [file ehpm-28-076-s001.png]
