# Supplementary material for: Associations of reproductive risk score and joint exposure to ambient air pollutants with chronic obstructive pulmonary disease: a cohort study in UK Biobank
Source: Environ Health Prev Med. 2023 Dec 7;28:76. doi: 10.1265/ehpm.23-00155 (PMC10711373; doi:10.1265/ehpm.23-00155)
Supplement: Supplementary file 2 — Additional file 2: Table S1. HRs (95% CI) for covariates in the multivariable adjusted Cox regression model. Table S2. Stratified analysis on incident COPD risk by APS tertiles in UK Biobank. Table S3. AUC and IDI of COPD according to individual items of and total RRS. [file ehpm-28-076-s002.docx]

**Table S1. HRs (95% CI) for covariates in the multivariable adjusted Cox regression model.**

| Covariates | RRS groups | PM_2.5_ tertiles | PM_2.5-10_ tertiles | PM_10_ tertiles | NO_2_ tertiles | NO_X_ tertiles | APS tertiles |
| --- | --- | --- | --- | --- | --- | --- | --- |
| Age | 1.09 (1.07-1.10) | 1.08 (1.07-1.10) | 1.08 (1.06-1.10) | 1.08 (1.06-1.10) | 1.08 (1.06-1.10) | 1.08 (1.07-1.102) | 1.08 (1.06-1.10) |
| BMI | 1.02 (1.01-1.03) | 1.02 (1.01-1.03) | 1.02 (1.01-1.03) | 1.02 (1.01-1.03) | 1.02 (1.01-1.03) | 1.02 (1.01-1.03) | 1.02 (1.01-1.03) |
| Education |  |  |  |  |  |  |  |
| None of the above | Ref. | Ref. | Ref. | Ref. | Ref. | Ref. | Ref. |
| Work-related practical qualifications | 1.14 (0.92-1.41) | 1.15 (0.93-1.43) | 1.15 (0.93-1.42) | 1.15 (0.93-1.42) | 1.16 (0.94-1.43) | 1.16 (0.93-1.43) | 1.16 (0.94-1.43) |
| Lower secondary education | 0.66 (0.58-0.76) | 0.67(0.58-0.77) | 0.65 (0.57-0.75) | 0.66 (0.57-0.76) | 0.67 (0.58-0.77) | 0.66 (0.58-0.77) | 0.67 (0.58-0.77) |
| Upper secondary education | 0.48 (0.38-0.60) | 0.48 (0.38-0.61) | 0.47 (0.37-0.60) | 0.48 (0.38-0.60) | 0.48 (0.38-0.61) | 0.48 (0.38-0.61) | 0.48 (0.38-0.61) |
| Higher education | 0.48 (0.41-0.56) | 0.49 (0.42-0.57) | 0.48 (0.41-0.56) | 0.48 (0.41-0.56) | 0.48 (0.41-0.56) | 0.49 (0.42-0.57) | 0.49 (0.41-0.57) |
| Employment |  |  |  |  |  |  |  |
| In paid employment or self-employed | Ref. | Ref. | Ref. | Ref. | Ref. | Ref. | Ref. |
| Not in paid employment | 2.03 (1.66-2.49) | 2.10 (1.72-2.57) | 2.10 (1.72-2.57) | 2.11 (1.72-2.58) | 2.09 (1.71-2.56) | 2.11 (1.72-2.57) | 2.11 (1.72-2.57) |
| Retired | 1.35 (1.16-1.57) | 1.35 (1.16-1.57) | 1.34 (1.15-1.56) | 1.36 (1.17-1.58) | 1.36 (1.17-1.59) | 1.35 (1.16-1.57) | 1.36 (1.16-1.58) |
| Smoking status |  |  |  |  |  |  |  |
| Never | Ref. | Ref. | Ref. | Ref. | Ref. | Ref. | Ref. |
| Current | 13.67 (11.70-15.98) | 14.17 (12.13-16.54) | 14.47 (12.40-16.89) | 14.22 (12.18-16.61) | 13.92 (11.92-16.26) | 14.19 (12.15-16.57) | 14.01 (12.00-16.37) |
| Previous | 3.28 (2.84-3.79) | 3.34 (2.89-3.86) | 3.36 (2.91-3.89) | 3.35 (2.90-3.87) | 3.33 (2.88-3.85) | 3.34 (2.89-3.86) | 3.33 (2.88-3.85) |
| Healthy alcohol intake^a^ | 0.76(0.68-0.85) | 0.75 (0.67-0.85) | 0.75 (0.67-0.84) | 0.75 (0.67-0.84) | 0.75 (0.67-0.85) | 0.75 (0.67-0.84) | 0.75 (0.67-0.85) |
| Healthy diet^b^ | 0.82 (0.69-0.97) | 0.82 (0.69-0.97) | 0.82 (0.70-0.97) | 0.8 (0.69-0.97) | 0.82 (0.70-0.97) | 0.82 (0.70-0.97) | 0.82 (0.70-0.97) |
| Healthy physical activity level | 0.72 (0.64-0.81) | 0.72 (0.64-0.81) | 0.72 (0.64-0.81) | 0.72 (0.64-0.80) | 0.72 (0.64-0.80) | 0.72 (0.64-0.81) | 0.72 (0.64-0.81) |
| ^a^ Healthy alcohol intake: 0<women≤14g/day. ^b^ Healthy diet: meeting 4-5 ideal food groups. | | | | | | | |
| Abbreviation: HR, hazard ratio; CI, confidence interval; RRS, reproductive risk score; PM_2.5_, particulate matter with aerodynamic diameter ≤2.5 μm; PM_2.5–10_, particulate matter with an aerodynamic diameter between 2.5 and 10 μm; PM_10_, particulate matter with aerodynamic diameter ≤10 μm; NO_2_, nitrogen dioxide; NO_x_, nitrogen oxide; APS, air pollution score; BMI, body mass index. | | | | | | | |

**Table S2. Stratified analysis on incident COPD risk by APS tertiles in UK Biobank.**

| **Air Pollution Score (tertiles)** | **Reproductive risk score groups** | COPD/N (%) | HR (95% CI) | |
| --- | --- | --- | --- | --- |
|  |  |  | Crude | Multivariable adjusted^a^ |
| Q1 | The low risk RRS (0-1) | 43/4,310 (1.00) | Ref. | Ref. |
|  | The low-mediate RRS (2-3) | 159/13,969 (1.14) | 1.14 (0.82, 1.60) | 1.29 (0.90, 1.85) |
|  | The high-mediate risk RRS (4-5) | 101/6,327 (1.60) | 1.61 (1.12, 2.29) | 1.68 (1.15, 2.46) |
|  | The high risk RRS (6-13) | 35/1,403 (2.49) | 2.55 (1.63, 3.98) | 2.29 (1.42, 3.69) |
|  | Per score increment |  | 1.17 (1.10, 1.25) | 1.14 (1.06, 1.22) |
| Q2 | The low risk RRS (0-1) | 49/4,249 (1.15) | Ref. | Ref. |
|  | The low-mediate RRS (2-3) | 198/13,636 (1.45) | 1.27 (0.93, 1.73) | 1.27 (0.92, 1.76) |
|  | The high-mediate risk RRS (4-5) | 123/6,577 (1.87) | 1.64 (1.18, 2.28) | 1.50 (1.06, 2.12) |
|  | The high risk RRS (6-13) | 53/1,547 (3.43) | 3.01 (2.04, 4.44) | 2.40 (1.59, 3.62) |
|  | Per score increment |  | 1.21 (1.14, 1.28) | 1.15 (1.08, 1.22) |
| Q3 | The low risk RRS (0-1) | 58/3,657 (1.59) | Ref. | Ref. |
|  | The low-mediate RRS (2-3) | 260/12,815 (2.03) | 1.29 (0.97, 1.71) | 1.21 (0.90, 1.63) |
|  | The high-mediate risk RRS (4-5) | 207/7,645 (2.71) | 1.73 (1.29, 2.32) | 1.55 (1.14, 2.11) |
|  | The high risk RRS (6-13) | 75/1,892 (3.96) | 2.56 (1.82, 3.61) | 2.00 (1.39, 2.89) |
|  | Per score increment |  | 1.20 (1.15, 1.26) | 1.15 (1.10, 1.21) |
| *P* for interaction^b^ |  |  | 0.171 | 0.086 |
| ^a^ Adjusted for age, body mass index; education, employment, smoking status, alcohol drinking, healthy diet status and physical activity.  ^b^ Tested by the likelihood-ratio test comparing models with and without the cross-product interaction term.  Abbreviation: COPD, chronic obstructive pulmonary disease; APS, air pollution score; RRS, reproductive risk score; HR, hazard ratio; CI, confidence interval. | | | | |

**Table S3. AUC and IDI of COPD according to individual items of and total RRS.**

| Items | Area under ROC curve ^a^ | IDI % (95% CI) ^b^ | *P* value |  |
| --- | --- | --- | --- | --- |
|  |  |  |  |  |
| SE1A | 0.804 | 0.30 (0.18-0.41) | <0.001 |  |
| FH1 | 0.805 | 0.26 (0.15-0.37) | <0.001 |  |
| FH2A | 0.805 | 0.17 (0.08-0.27) | <0.001 |  |
| FH3 | 0.805 | 0.20 (0.10-0.31) | <0.001 |  |
| FH3A | 0.804 | 0.30 (0.19-0.41) | <0.001 |  |
| FH3C | 0.804 | 0.26 (0.15-0.38) | <0.001 |  |
| FH3D | 0.804 | 0.31 (0.19-0.42) | <0.001 |  |
| FH4* | 0.804 | 0.22 (0.12-0.32) | <0.001 |  |
| FH5* | 0.804 | 0.30 (0.19-0.41) | <0.001 |  |
| FH9* | 0.805 | 0.20 (0.12-0.29) | <0.001 |  |
| FH10* | 0.804 | 0.28 (0.18-0.39) | <0.001 |  |
| Total (RRS) | 0.808 |  |  |  |
| ^a^ Logitic regression were conducted by adjusting for age, body mass index, education, employment, smoking status, alcohol drinking, healthy diet status and physical activity.  ^b^ Integrated discrimination improvement (95% CI) were calculated in reproductive score compared with single items, and adjusted for age, body mass index, education, employment, alcohol drinking, smoking status, healthy diet status and physical activity.  *FH4 were included FH4A, FH4B and FH4C; FH5 were included FH5A; FH9 were included FH9A; FH10 were included FH10A.  Abbreviation: AUC, area under curve; COPD, chronic obstructive pulmonary disease; RRS, reproductive risk score; ROC, receiver operating characteristic curve; IDI, integrated discrimination improvement; CI, confidence interval. | | | |  |
